# Prenatal Tobacco Exposure and Behavioral Disorders in Children and Adolescents: Systematic Review and Meta-Analysis

**DOI:** 10.3390/pediatric16030062

**Published:** 2024-08-31

**Authors:** Stephanie Godleski, Shannon Shisler, Kassidy Colton, Meghan Leising

**Affiliations:** 1 Department of Psychology, College of Liberal Arts, Rochester Institute of Technology, Rochester, NY 14623, USA; 2 Clinical and Research Institute on Addictions, State University of New York at Buffalo, Buffalo, NY 14203, USA; smcasey@buffalo.edu (S.S.); mlcasey@buffalo.edu (M.L.); 3 Department of Psychology, School of Arts and Sciences, University of Rochester, Rochester, NY 14627, USA; kcolton@ur.rochester.edu

**Keywords:** prenatal tobacco exposure, attention deficit/hyperactivity disorder, oppositional defiant disorder, conduct disorder

## Abstract

Prenatal tobacco exposure has been implicated in increased risk of the development of behavioral disorders in children and adolescents. The purpose of the current study was to systematically examine the association between prenatal tobacco exposure and diagnoses of Attention Deficit/Hyperactivity Disorder, Oppositional Defiant Disorder, and Conduct Disorder in childhood and adolescence. We searched Medline, Psychinfo, ERIC, Proquest, Academic Search Complete, PsychArticles, Psychology and Behavioral Sciences Collection, Web of Science, CINAHL Plus, and Google Scholar databases through October 2022. The authors screened studies and extracted data independently in duplicate. Ten clinical studies examining diagnoses of Attention Deficit/Hyperactivity Disorder, Oppositional Defiant Disorder, and Conduct Disorder between the ages of 4 and 18 years old were included. There was insufficient evidence to synthesize outcomes related to Conduct Disorder and Oppositional Defiant Disorder. The meta-analysis found a significant effect of prenatal tobacco exposure in increasing the likelihood of an Attention Deficit/Hyperactivity Disorder diagnosis in childhood and adolescence. Implications for future research are discussed.

## 1. Introduction

Maternal smoking during pregnancy continues to be a public health concern, as tobacco is commonly used during pregnancy with rates of tobacco use during pregnancy remaining stable [1,2]. Indeed, 5.5% of infants born in 2020 were prenatally exposed to tobacco [3]. Therefore, understanding the risk that prenatal tobacco exposure poses for later developmental sequelae is critical. Prenatal tobacco exposure (PTE) is associated with a higher risk of a variety of negative physical, cognitive, and socio-behavioral health outcomes for children both early and later in development, including restricted fetal growth, poor self-regulation, learning and memory deficits, obesity, and aggressive behavior [4,5,6,7,8,9,10]. Indeed, prenatal tobacco exposure has been suggested to impact brain function, physiological dysregulation, and early neurobehavioral outcomes [11,12].

In particular, prenatal tobacco exposure is implicated in increased impulsivity, dis-inhibition, dysregulation, activity, and inattention [6,7,13,14] and, thus, a higher risk of the development of attentional, externalizing, and disruptive behavior disorders [15,16]. Specifically, prenatal tobacco exposure has been linked to behavioral disorders (BDs), including Attention Deficit/Hyperactivity Disorder (ADHD), Oppositional Defiant Disorder (ODD), and Conduct Disorder (CD) [17,18,19,20,21,22]. This suite of disorders is characterized by hyperactivity, inattention, impulsivity, and defiant behavior including noncompliance with limit-setting by adults or peers and hostile or aggressive physical behavior towards others. 

Importantly, BDs can result in many challenges for affected children. They may have difficulty forming positive relationships with peers and adults and may be more likely to have problems with bullying and victimization in school [23,24]. Other adverse outcomes include poor academic achievement, higher odds of substance use disorders, unemployment, and criminal behavior [25]. In addition, BDs are often comorbid with other mental health disorders (e.g., anxiety) [26,27]. Diagnoses such as ADHD, ODD, and CD can also often bear considerable financial and emotional burdens for individuals, families and caregivers, agencies, and society as a whole. BD diagnoses are also associated with increased caregiver strain and diminished parental mental health [28,29,30,31]. Additionally, childhood BDs are estimated to produce expenses upwards of $52.4 billion a year, including costs associated with hospitalizations, outpatient visits, prescriptions, detention centers, medically related work absences for parents, and education [28,32,33,34,35]. Finally, CD and ADHD carry a significant global health burden, with estimates of 5.75 million years lived with disability (YLDs) for CD, and 491,500 YLDs for ADHD, accounting for 0.8% of YLDs globally [36]. YLDs reflect the extent to which a disability impacts quality of life with one YLD equivalent to one year of healthy life lost due to disability. Thus, it is important to understand the potential for increased risk of these disorders due to prenatal tobacco exposure. 

The magnitude of the association between prenatal tobacco exposure and diagnosed BDs is unclear, as much research examines the symptoms or facets of disruptive behavior or externalizing behavior in general (e.g., hyperactivity, inattention, impulsivity) instead of clinical diagnoses, and the results are mixed [37,38,39,40,41,42]. Understanding the impact of prenatal tobacco exposure on symptomatology that meets the threshold for diagnoses is an important extension of past work and is likely to be especially informative for understanding clinically significant levels of BD rather than prenatal tobacco exposure’s impact on BD symptoms alone [43]. In order to receive a clinical diagnosis, individuals must present with both a number of symptoms and functional impairment [44]. Thus, a clinical diagnosis is naturally concomitant with functional impairment, whereas counts of BD symptoms may or may not reflect clinically significant levels of behavioral concerns or be associated with significant impaired functioning [45]. Indeed, to some extent, externalizing symptoms are not uncommon, particularly in younger children, such that these behaviors may be developmentally typical in early childhood. What is most worrying is when the persistence or severity of the symptoms impedes the ability of the child to function well in their environment. Therefore, in addition to past work that has examined the impact of prenatal tobacco exposure on BD symptomatology on a continuum, it is also important to understand the impact of exposure in predicting more severe, impairing levels of BD that meet a clinical threshold, given the significant wide-reaching impact of diagnosed BDs [36]. In examining prenatal tobacco exposure specifically regarding the likelihood of BD diagnoses, we may better understand how smoking during pregnancy impacts overall daily functioning in addition to symptomatology [46]. Therefore, the present systematic review and meta-analysis fills a critical gap in the literature regarding the relationship between prenatal tobacco exposure and disruptive, externalizing behavior that meets the threshold for BD diagnoses.

Most previous reviews have synthesized adjusted associations between prenatal to-bacco exposure and BD diagnoses or externalizing behavior problems; however, there is a lack of consistency across studies in what confounding variables are adjusted for analytically, which has been argued to make it difficult to interpret the summary estimates of prenatal exposure on outcomes such as ADHD [43,47,48]. Indeed, much of the previous research has adjusted for a variety of sociodemographic variables (e.g., socio-economic status, maternal age, maternal education) when examining the link between prenatal tobacco exposure and BDs; however, many studies also take into account other potential influences or confounding variables such as prenatal exposure to other substances or maternal mental health [47,48,49,50]. The constellation of influences that are adjusted for varies significantly from study to study (e.g., different sociodemographic risk factors, parental mental health concerns, pregnancy and birth outcomes) [50]. This lack of consistency in included potential covariates produces adjusted associations that are not comparable, impacting the ability to combine them via meta-analysis [51]. Thus, the current synthesis focused on investigating unadjusted associations between exposure and subsequent diagnoses, and expands on previous work by examining the unadjusted relationship between tobacco exposure and focusing on clinical diagnoses, which may reflect a greater degree of severity and impairment. Therefore, the purpose of the current study was to systematically examine the association between prenatal tobacco exposure and the BD diagnoses of ADHD, ODD, and CD between the ages of 4 and 18 years old. The age ranges were chosen to reflect pre-school or school-aged children and adolescents. Further, the validity of diagnoses of ADHD, ODD, and CD have been supported at preschool age and diagnoses of such disorders as early as the preschool period have shown stability into the school age years, whereas diagnoses prior to the preschool period may be less valid and stable [52,53].

## 2. Materials and Methods

### 2.1. Selection of Studies

Studies included in this synthesis were obtained from several sources. First, we completed electronic searches of Medline, Psychinfo, ERIC, Proquest, Academic Search Complete, PsychArticles, Psychology and Behavioral Sciences Collection, Web of Science, CINAHL Plus, and Google Scholar in October 2022. We imposed no date restrictions on our search. We searched for both published and unpublished manuscripts, such as dissertations and theses, in order to reduce the possibility of publication bias [54]. The first part of the search term indicated the developmental period and substance, thus included all iterations of the following: *pregnancy* or *prenatal* or *maternal* with *smoking* or *nicotine* or *tobacco* or *cigarette*. This was paired with a key word indicating our outcome of interest, for which we used attention, inattention, impulsivity, attention deficit hyperactivity disorder, attention deficit disorder, ADHD, externalizing behavior, conduct, conduct disorder, conduct problems, oppositional, oppositional defiant disorder, disruptive behavior disorder, disruptive behav*, or hyperactivity.

### 2.2. Data Analysis Plan

We fit a random-effects model to the data and estimated the amount of heterogeneity (i.e.,*τ*^2^), using the DerSimonian–Laird estimator [55]. In addition, we report the *Q*-test for heterogeneity and the *I*^2^ statistic [56,57]. We use studentized residuals and Cook’s distances to examine whether studies may be outliers and/or influential in the context of the model [58]. We consider studies potential outliers if they have a studentized residual larger than the 100 × (1 − 0.05/(2 × *k*))th percentile of a standard normal distribution (i.e., using a Bonferroni correction with two-sided α = 0.05 for *k* studies included in the meta-analysis). The analysis was carried out using R (version 4.0.0) and the metafor package (version 2.5.82) [59,60].

### 2.3. Publication Bias

Given that statistically significant results are more likely to be published than non-significant results, any meta-analysis should take the potential for publication bias into account [61]. We searched for both published and unpublished manuscripts (i.e., unpublished dissertations and theses) as one strategy to decrease the likelihood of finding publication bias. The rank correlation test and the regression test, using the standard error of the observed outcomes as a predictor, are used to check for funnel plot asymmetry, but it is necessary to have at least 10 studies in an analysis for tests of publication bias to be valid, so we were only able to assess publication bias for effects of prenatal exposure on ADHD diagnosis in the current review [62,63].

### 2.4. Quality Assessment

We assessed the risk of bias in the included studies by drawing on the signaling questions in the checklist for cohort studies appraisal tool from the Joanna Briggs Institute (JBI) [64]. The items in this checklist cover the methodological quality of the research to help determine if it was adequately protected from potential sources of bias. Two reviewers undertook the risk of bias assessment independently, and any disagreements were resolved by consensus, or by a third reviewer if necessary. We assessed the risk of bias at the paper level, coding each paper on the 11 criteria covered by the tool. Response options were “Yes”, “No”, “Unclear”, and “Not applicable”, with yes answers indicating the lowest risk of potential bias. We report the results of the assessment for each of the assessed criteria for each included study.

## 3. Results

### 3.1. Search Results, Quality Assessment, and Coding Features 

#### 3.1.1. Search Outcomes

**Figure 1:**
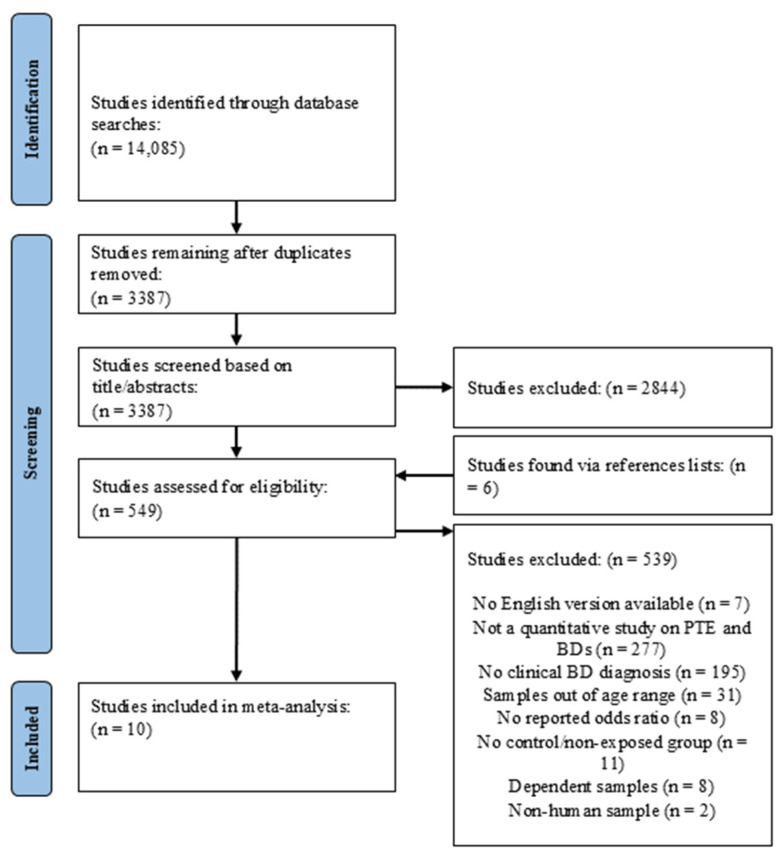
Identification of studies via databases and registers.

Searches yielded 14,085 references for consideration. After removing duplicate references, a total of 3387 manuscripts remained from the initial literature search. The initial screening involved reviewing the titles and abstracts of all 3387 studies, and eliminated articles that were not quantitative, were conducted solely with animals, or did not include a measure of prenatal tobacco exposure and a diagnosed BD. This initial screening allowed for the direct exclusion of 2844 manuscripts, and the texts of the remaining 543 manuscripts were read in full (see Figure 1). Additional studies were collected by conducting a snowball search of the reference lists of included studies and relevant meta-analyses, which generated an additional 6 articles for consideration which were also read in full. 

#### 3.1.2. Study Inclusion Criteria

The studies that were selected for inclusion quantified the relationship between prenatal tobacco exposure and diagnosed BDs in children aged 4 to 18 years old. We excluded studies from the meta-analysis if they (a) were not written in English (e.g., [65,66]); (b) were not a quantitative study of the relationship between prenatal tobacco exposure and BDs (e.g., [67,68]); (c) did not include a clear measure of a clinically diagnosed BD (e.g., [37,69]); (d) did not include a non-exposed comparison/control group that did not smoke during pregnancy (e.g., [70,71,72,73]); (e) had samples that were either less than 4 years of age, or greater than 18 years of age or included children that were below 4 or above 18 (e.g., [74,75,76]) or did not report child age at the time of diagnosis [77]; (f) did not report an unadjusted odds ratio, or information that could be meaningfully converted to an unadjusted odds ratio, such as a 2 × 2 frequency table that included the number of exposed vs. non-exposed participants and number diagnosed and not diagnosed within those categories (e.g., [78,79,80,81,82]); (g) had a sample that was not clearly independent (e.g., twin sample, large national samples covering several years of births where siblings were not clearly excluded [83,84,85]).

#### 3.1.3. Studies Sharing Common Data

One of the included studies reported on more than one BD [86]. Since we were underpowered for a robust variance analysis and only one study reported exclusively on ODD and CD diagnoses, we chose to run a separate analysis for ADHD, in order to create independence among the effects. Nigg and Breslau (2007) was the only study that reported across multiple waves and included diagnoses at several age ranges within the same longitudinal sample; we chose the timing of the wave (i.e., child age) based on examining a similar developmental period as the other included studies—to the closest approximation [86]. Therefore, for Nigg and Breslau (2007), we chose to include the effect sizes from the 6-year-old wave for analyses to be consistent with the majority of the other studies included [86]. Sagiv et al. (2013) was the only study to include maternal smoking categorized as a heavier use (i.e., >10 cigarettes/day), a lighter use (1–10 cigarettes/day), and a non-smoking group, and we chose to include the heavier use group in comparison to the non-smoking group [87]. Huang et al. (2019) reported on two independent samples with two independent effect sizes; therefore, both were included for the purposes of the current review [88]. In the end, we included a total of 10 studies with 11 independent samples in this review, contributing a total of 13 effect sizes (11 for ADHD, 1 for ODD, and 1 for CD). 

The typical study in this review reported on the relationship between prenatal tobacco exposure and BDs in children using some form of data that allowed for calculation of an odds ratio. Most studies reported on a combination of ages or grade levels, most typically spanning from middle childhood through adolescence. In the typical study, prenatal tobacco exposure was maternally self-reported, and a standardized diagnostic interview was used to measure clinical diagnoses of BDs.

#### 3.1.4. Risk of Bias Assessment

The risk of bias assessment for each domain for each included study is presented in Table 1. The most common source of potential bias was in the measurement of exposure. Most studies used retrospective self-reports of pregnancy smoking, which may be susceptible to both recall and social desirability bias. No studies used prospective biological assays of tobacco exposure, nor did any use well-validated methods for capturing daily substance use patterns (e.g., substance use assessment interview, biological assay during pregnancy). In addition, most authors did not explore the extent of potential selection bias. Only one study presented a balance table of differences between exposure groups on baseline measures of demographics. Finally, there was a general lack of clarity around attrition and missing data. Often, large portions of the target sample were missing from analyses with no explanation and no analysis of whether missing data were differential between exposure groups.

#### 3.1.5. Coding Study Features

To ensure sufficient reliability of the data, two coders independently reviewed and coded all studies included in our synthesis. Inter-rater reliability was computed separately for each variable as the percentage of agreement between coders. These reliabilities ranged from 75% for socio-economic status to 100% for publication year. Low reliabilities for socio-economic status were the result of coders’ differing interpretations of the information presented on socio-economic status (i.e., whether the information was clear or if inferences could be drawn from the information provided), and these were resolved quickly. All coding disagreements were resolved through discussion and consensus prior to any data analyses. 

We coded several variables to be considered as possible moderators of the relationship between prenatal tobacco exposure and BD diagnoses (e.g., sample recruitment, location, sample size; sample demographics, behavior measure used/reporter, method of assessing prenatal tobacco exposure). However, there was variability in the nature of the reporting practices in the primary studies, reducing our ability to consider all of the potential moderators in our analyses and, as detailed subsequently, there was no significant amount of heterogeneity in the true outcomes suggesting that moderator analyses were not appropriate. Therefore, potential sources of variation in study findings were not examined. 

#### 3.1.6. Prenatal Tobacco Exposure

Prenatal tobacco exposure was typically reported as a binary occurrence. For example, most studies reported whether or not mothers smoked during their pregnancy. Mothers self-reported their prenatal tobacco use in most of the studies.

#### 3.1.7. Behavior Disorders

The assessment of BD used was coded for all studies (see Table 2). Five of the studies included assessed BD via clinical/hospital diagnoses based on criteria from the Diagnostic and Statistical Manual of Mental Disorders (Third Edition, Revised, DSM-III-R; [95]; Fourth Edition, DSM-IV-TR, [96]; e.g., [88,90]). An additional four studies in this analysis reported using some form of structured diagnostic interview, such as the Diagnostic Interview Schedule ([97,98]; e.g., [89]) or the Schedule for Affective Disorders and Schizophrenia for School-Age Children (K-SADS; [99,100]; e.g., [94]). The final study assessed for BD diagnoses using the Multimodal Treatment Study for Attention-Deficit/Hyperactivity Disorder version of the Swanson, Nolan, and Pelham, Version IV Questionnaire (MTA-SNAP-IV; [101]; e.g., [89]).

#### 3.1.8. Effect Sizes

Effect sizes were recorded as unadjusted odds ratios. When odds ratios were not presented in the manuscript, the information from the 2 × 2 frequency table was inputted into the Campbell Effect Size Calculator [102]. Odds ratios were converted to log odds and the variance of the log odds were computed for use in the analysis (this is required to maintain symmetry in the analysis). The summary effect sizes were then converted back into odds ratios for interpretation.

### 3.2. Effects of Prenatal Tobacco Exposure on BD Diagnoses

#### 3.2.1. Effects of Prenatal Tobacco Exposure on ADHD Diagnosis

**Figure 2:**
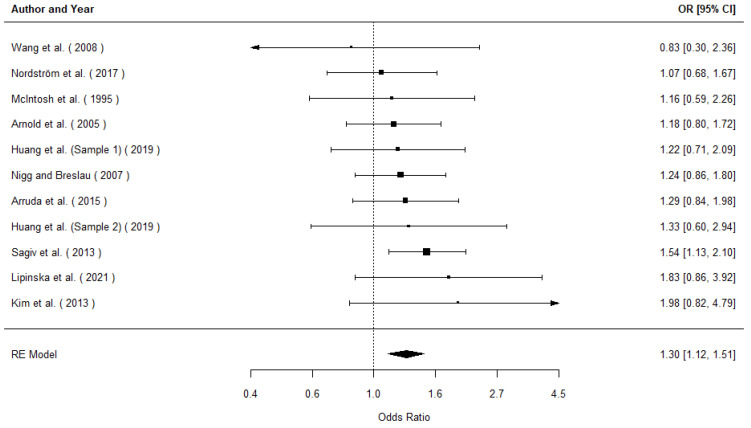
Forest plot of the random effects model of the relationship between prenatal tobacco exposure and ADHD diagnosis [39,86,87,88,89,90,91,92,93,94].

**Figure 3:**
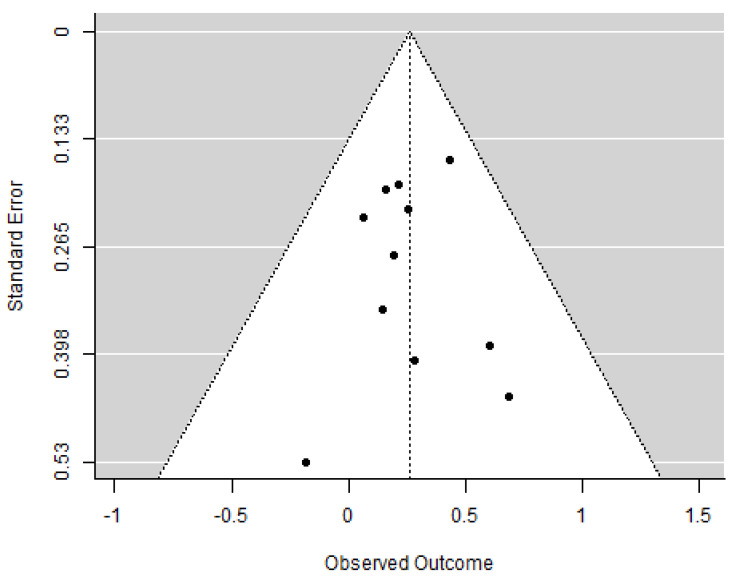
Funnel plot for ADHD. Note. Dots represent the observed outcomes in the included studies.

We included a total of *k* = 11 independent effects in the analysis, including 1402 exposed children and 6383 non-exposed children (for a total of 7785). The observed outcomes expressed as log odds ranged from −0.18 to 0.69. The estimated average outcome based on the random-effects model was $\hat{\mu }$ = 0.26 (95% CI: 0.12 to 0.41). Therefore, the average outcome differed significantly from zero (*z* = 3.49, *p* < 0.001), indicating that children prenatally exposed to tobacco were significantly more likely to be diagnosed with ADHD. A forest plot showing the observed outcomes and the estimate based on the random-effects model (converted from log odds to odds ratio) is shown in Figure 2. According to the *Q*-test, there was no significant amount of heterogeneity in the true outcomes (*Q*(10) = 4.75, *p* = 0.91, ${\hat{\tau }}^{2}$= 0.00, *I*^2^ = 0.00%); thus, moderator analyses were not appropriate. One study—Sagiv et al. (2013)—had a relatively large weight compared to the rest of the studies and could be considered overly influential [87]. However, if Sagiv et al. (2013) is removed from the analysis, the result is only slightly lower (OR = 1.24) and remains statistically significant (*p* = 0.01) [87]. An examination of the studentized residuals revealed that none of the studies had a value larger than ±2.84 and hence there was no indication of outliers in the context of this model. A funnel plot of the estimates is shown in Figure 3. Neither the rank correlation nor the regression test indicated any funnel plot asymmetry (*p* = 1.000 and *p* = 0.899), indicating that no publication bias is detected.

#### 3.2.2. Effects of Prenatal Tobacco Exposure on ODD Diagnosis

One study (Nigg and Breslau, 2007) examined the relationship between prenatal tobacco exposure and ODD diagnosis [86]. Their study, conducted in the US, utilized a sample of 798 17-year-old children. They found that prenatally exposed children were 2.19 times more likely to be diagnosed with ODD than non-exposed children [95% CI: 1.40 to 3.45]. 

#### 3.2.3. Effects of Prenatal Tobacco Exposure on CD Diagnosis

Nigg and Breslau (2007) were also the only authors to report on the relationship between prenatal tobacco exposure and diagnosis of conduct disorder [86]. They found that children prenatally exposed to tobacco were 2.19 times more likely to have been diagnosed with conduct disorder than unexposed children (OR 2.19, [95% CI: 1.21 to 3.97]). 

## 4. Discussion

The current meta-analyses aimed to examine the association between prenatal tobacco exposure and diagnoses of ADHD, ODD, and CD in childhood and adolescence. Based on the 10 studies reviewed examining child and adolescent ADHD diagnoses, children prenatally exposed to tobacco were significantly more likely to be diagnosed with ADHD. These results are consistent with previous reviews demonstrating associations between prenatal tobacco exposure with disruptive behavior and externalizing symptoms [21,50,103,104,105] and adds by synthesizing the literature on tobacco exposure and specifically clinical diagnoses as an outcome. Only one study reviewed exclusively reports on ODD and CD diagnoses as an outcome (i.e., [86]). While Nigg and Breslau (2007) did find that children prenatally exposed to tobacco were significantly more likely to be diagnosed with both ODD and CD, in the absence of more evidence we cannot make strong conclusions regarding either relationship [86]. However, past work has suggested a particularly strong relationship between maternal smoking during pregnancy and CD symptomatology (e.g., [106]). The BDs are often comorbid, including approximately 50% of those diagnosed with ADHD within the study by Arnold et al. (2005) who were also diagnosed with ODD or CD [89]. Future research examining the direct association between prenatal tobacco exposure and diagnosed ODD and conduct problems, as well as those with comorbid diagnoses of more than one BD, would allow for further synthesis work. Despite differences in the ways the included studies approached examination of the relationship between prenatal tobacco exposure and BDs, there was no significant heterogeneity in the analyses for ADHD. Given the homogeneity, we did not examine potential sources of variation. The forest plot in Figure 1 illustrates the consistency of the effect sizes across the body of evidence for ADHD.

These findings help to clarify previous research focusing on symptoms or facets of behavioral disorders and demonstrate the impact of prenatal tobacco exposure for increasing the likelihood of diagnoses of ADHD, in particular. The current synthesis demonstrates the direct association between prenatal tobacco exposure and later clinically significant diagnoses of BD. Future research would benefit from examining the mechanisms driving the association between prenatal tobacco exposure and later diagnoses of attentional or behavioral problems. Prenatal tobacco exposure can have teratogenic effects on attention and regulation systems (e.g., atypical nervous system development; [5,15,107,108,109]), which is evident in more highly aroused and reactive neonatal behaviors [110,111] and is associated with the later development of behavioral problems (e.g., [112]). Therefore, one mechanism through which prenatal tobacco exposure may be associated with attentional and disruptive behavior disorders is through the teratological impact of the tobacco exposure itself (e.g., [106]). In addition, shared genetic risk between parents and offspring may be an important factor for BDs [21,113]; however, genetically informed studies have also suggested the important influence of family or the social environment in the association between prenatal tobacco exposure and behavior problems [114]. For example, maternal smoking during pregnancy is also associated with higher rates of maternal psychopathology (e.g., [115,116]) as well as with less effective parenting practices (e.g., harsh discipline, less nurturing and sensitive; [117,118,119,120]). Such parenting practices may increase the risk of disinhibition, dysregulation, and behavioral problems (e.g., [13,115]), while maternal responsiveness in the context of prenatal tobacco exposure may be protective (e.g., [13]). Therefore, parent–child interactions and parenting practices may also serve as a potential moderator of the association between prenatal tobacco exposure and BDs.

### Limitations

The current synthesis included 10 studies with 11 total independent samples, with only 1 study examining ODD and CD, which limits the generalizability of our findings for those outcomes. In addition, although the results suggested that there was not a significant amount of heterogeneity in the true outcomes for ADHD, there may have been limited power to detect heterogeneity due to the small sample size of the studies. An age range encompassing preschool- or school-aged through adolescence was chosen to represent school-aged children and adolescents and reflected the age ranges within many of the reviewed studies that spanned from preschool and kindergarten ages (4 or 5 years old) to mid to late adolescence (12 to 18 years old). There can be significant changes across these developmental periods and although past research has supported the stability of diagnoses as early as preschool-aged (e.g., [53]), there is the possibility that diagnoses may not persist from earlier to later periods. Further, the dose and timing of exposure may be significant factors influencing child outcomes [47,50,82]. Importantly, there was variation across reviewed studies in the detail with which prenatal tobacco exposure was assessed, ranging from a dichotomized approach (i.e., yes/no) and categorizing prenatal tobacco exposure (e.g., exposed versus non-exposed) to considering the level or quantity of smoking [87]. However, exposure group statuses across the reviewed studies were based primarily on maternal self-report of use. Patterns of smoking can vary across pregnancy [106,121], and methodology for assessing tobacco exposure may be important to consider [106,122]. In particular, reliance on solely maternal self-report of use is a significant limitation of past research, especially when quantified as a dichotomy of either exposed vs. not exposed without consideration of dose and timing of prenatal tobacco exposure across the prenatal period. 

Importantly, the current review adds to previous research by focusing on studies that reported unadjusted associations between prenatal tobacco exposure and BD diagnoses in order to pool across comparable associations of the direct relationship [51]. Given that much of the previous research has covaried for a wide variety of factors associated with smoking during pregnancy, unadjusted associations allow for a clearer synthesis of the direct relationship between prenatal tobacco exposure and BDs. Smoking during pregnancy may be a marker of other risks for children developing externalizing behavior problems, including lower-quality parent–child interactions (e.g., Eiden et al., 2023), or shared genetic vulnerability [123]. The findings of the current review demonstrated pooled associations of a similar magnitude between prenatal tobacco exposure and ADHD as previous reviews examining ADHD symptomatology (e.g., pooled adjusted associations ranging from 1.56 to 1.64, [47,48,49,50]; unadjusted associations of 1.76, [49]). Across these meta-analyses and the current meta-analysis, the direct influence of prenatal tobacco exposure on BDs regardless of consideration of covariates is apparent. Nevertheless, there may be an important role of potential covariates, such as parental BD diagnoses or other substance exposure. For example, prenatal tobacco smokers are more likely to also use other substances, such as cannabis [124]. This comorbidity was not examined in the current study as very few of the reviewed studies examined cannabis exposure, but it may be useful for future research to consider potential additive or multiplicative effects. Alcohol use may also be an important additional substance to consider given that past meta-analyses examining ADHD symptomatology have suggested the importance of considering the impact of maternal alcohol use [50]. Adjusting for parental ADHD, however, has produced comparable associations in comparison to studies that did not adjust for parental ADHD [50]. Finally, we completed a quality assessment and recognized that due to the phenomenon under study and the methods through which prenatal tobacco exposure was typically assessed (i.e., maternal self-report), no study was in the low risk of bias category due to potential selection and reporting biases.

## 5. Conclusions

Based on the studies reviewed in this meta-analysis, there was a significant effect of prenatal tobacco exposure in increasing the likelihood of an ADHD diagnosis in childhood and adolescence. Although prenatal tobacco exposure does appear to be a risk factor for later ADHD diagnoses, the causal pathways linking the two are unclear and require further examination, such as teratological and familial influences. It may also be important for future studies to adopt consistent criteria when measuring prenatal tobacco exposure and clinical diagnoses to provide a more conclusive inference. Finally, it would be beneficial for additional research to include diagnoses of a broader spectrum of BD diagnoses more consistently, such as ODD and CD, as well as the potential comorbidities of the BDs.

## Figures and Tables

**Table 1:** Quality Assessment Ratings.

Assessment Domain	Groups Similar and from Same Population	Tobacco Exposure Measured Similarly	Tobacco Exposure Measure Valid and Reliable	Confounding Factors Identified	Strategies to Deal with Confounding Factors	Groups Free of BD at the Time of Exposure	BD Measure Valid and Reliable	Follow Up Time Reported and Sufficient	Follow Up Complete and Reasons for Loss to Follow Up Described/Explored	Strategies to Address Incomplete Follow Up Utilized	Appropriate Statistical Analysis Used
Arnold et al. (2005) [89]	+	+	−	+	+	+	+	+	−	−	+
Arruda et al. (2015) [39]	Unclear	+	−	+	Unclear	+	+	+	Unclear	−	+
Huang et al. (2019) [88]	−	+	−	+	+	+	−	+	+	N/A	+
Kim et al. (2013) [90]	−	+	−	+	+	+	−	+	+	N/A	+
Lipinska et al. (2021) [91]	−	+	−	+	−	+	+	+	Unclear	−	+
McIntosh et al. (1995) [92]	Unclear	+	−	+	+	+	+	+	+	N/A	+
Nigg and Breslau (2007) [86]	Unclear	+	−	+	+	+	+	+	Unclear	+	+
Nordström et al. (2017) [93]	Unclear	+	−	+	+	+	+	+	−	−	+
Sagiv et al. (2013) [87]	−	+	−	+	+	+	−	+	−	−	+
Wang et al. (2008) [94]	−	+	−	+	+	+	+	+	+	N/A	+

Note. + = Yes; − = No; N/A = Not applicable.

**Table 2:** Characteristics of studies included on prenatal tobacco exposure and BD diagnoses.

Study	Site	Number Exposed/Total Participants	Age (Years)	Assessment of Prenatal Smoking	BD Diagnosis Outcome	Assessment of BD
Arnold et al. (2005) [89]	United States and Canada	163/714	7–9.9	Baseline assessment asked mothers about smoking in gestational period (yes/no)	ADHD; 215/468 with ADHD had comorbid ODD/CD	Diagnostic Interview Schedule for Children (DISC-IV)
Arruda et al. (2015) [39]	Brazil	495/1830	5–13	Parents completed a study questionnaire	ADHD	Parents and/or caregivers completed the MTA-SNAP-IV scale
Huang et al. (2019) [88]	China	Discovery Sample: 56/1058 Stage One Sample: 26/674	6–18	Maternal questionnaire covering environmental risk factors including prenatal smoking (yes/no)	ADHD	Diagnosis of ADHD by psychiatrists using DSM-IV diagnostic criteria
Kim et al. (2013) [90]	United States	39/129	5–12	Parents completed a questionnaire about prenatal smoke exposure (yes/no) and other covariates	ADHD	A previous diagnosis by a physician based on DSM-IV diagnostic criteria
Lipinska et al. (2021) [91]	Poland	57/282	7–17	Mothers completed a pregnancy and perinatal history questionnaire, including prenatal smoking (yes/no)	ADHD	Diagnosis of ADHD using DSM-IV-TR or ICD-10 criteria, verified with a structured history questionnaire
McIntosh et al. (1995) [92]	United States	41/265	6–13	Mothers reported on smoking during pregnancy with the Maternal Perinatal Scale	ADHD	Diagnosis by physicians and psychologists of ADHD using DSM-III criteria, verified with school health and testing records
Nigg and Breslau (2007) [86]	United States	247/600 with prenatal exposure vs. never exposed	6–17	Mothers reported on their daily smoking habits during pregnancy during an interview (yes/no)	ADHD age 6; ODD age 6; ADHD age 11; ODD age 11; ODD age 17; CD age 17	Diagnostic Interview Schedule for Children (DISC; version 2.1) and Diagnostic Interview Schedule for Youth (DIS-Y)
Nordström et al. (2017) [93]	Finland	97/316 with ADHD diagnosis data	15–16	Information was systematically collected at antenatal clinics and birth hospital via self-report questionnaires and the delivery records	ADHD and/or DBD	At 15–16, Schedule for Affective Disorders and Schizophrenia for School-age Children—present and lifetime version (K-SADS-PL)
Sagiv et al. (2013) [87]	United States	166/560 with smoking data	8	Mothers reported on their prenatal smoking approximately two weeks after birth on a questionnaire	ADHD	Pediatric records were reviewed at the time of assessment for ADHD diagnoses
Wang et al. (2008) [94]	China	15/1260	4–12	Covariates and confounder assessed via clinical records or questionnaires completed by interviewing parents	ADHD	Schedule for Affective Disorders and Schizophrenia for School-age Children (K-SADS-E) modified to assess DSM-IV-R criteria

## Data Availability

Data are available upon reasonable request to the first author.
